# The clinical effects of ultrasound-guided microwave ablation in the treatment of primary hepatic carcinoma: Protocol for a retrospective clinical observation

**DOI:** 10.1097/MD.0000000000028045

**Published:** 2021-12-03

**Authors:** Bing Ran, Yong-Li Chang, Jing Qi, Wei Zhong, Min-Na Chen, Wang Zhang, Lin-Xia Meng

**Affiliations:** a The Second Affiliated Hospital of Shaanxi University of Chinese Medicine, Xianyang, Shaanxi, PR China; b The Baoji Hospital of Traditional Chinese Medicine, Baoji, Shaanxi, PR China.

**Keywords:** clinical trials, hepatocellular carcinoma, ultrasound-guided microwave ablation

## Abstract

**Instruction:**

Despite the continuous advancement of liver cancer diagnosis technology and level, there are still nearly two-thirds of patients with primary liver cancer that are already advanced at the time of diagnosis. Ultrasound-guided microwave ablation, as a palliative treatment for intermediate and advanced liver cancer, is currently recognized internationally Standard treatment for patients with unresectable hepatocellular carcinoma. However, recently, some scholars hold that ultrasound-guided microwave ablation does not guarantee complete inactivation of tumor lesions.

**Methods/design:**

This study will evaluate the safety and effectiveness of ultrasound-guided microwave ablation in patients with advanced hepatocellular carcinoma through retrospective analysis. This study will follow a clinical research method with consecutive enrollment. The overall survival rate, objective tumor remission rate, serum indices and incidence of adverse effects after treatment will be counted for patients.

**Discussion:**

At present, there are no good treatment options for intermediate and advanced hepatocellular carcinoma. Therefore, there is a strong demand to explore the individualized multidisciplinary combined treatment model based on ultrasound-guided microwave ablation.

**Trial registration:**

ClinicalTrials.gov, ChiCTR2100052107, Registered on 17 October 2021.

## 1 Instruction

Hepatocellular carcinoma (HCC) is one of the most common tumors in the world. The five-year overall survival rate of the patient is less than 12%.^[1–3]^ Research in China have shown that the fatality rate of HCC is second only to lung cancer, and it ranks second in the fatality rate of various cancers in China. Moreover, most HCC patients in China are in the middle and advanced stages of the disease when they see a doctor.^[4,5]^ The incidence of liver cancer is closely related to factors such as viral hepatitis, cirrhosis, family inheritance and certain chemical substances.^[6]^ In recent years, with the improvement of people's health awareness and the development of medical imaging technology, more and more people have new knowledge of HCC, and the early screening rate and diagnosis rate of HCC are also increasing. For the treatment of patients with HCC, surgical resection is the best treatment method in the early stage. However, HCC has an insidious onset and progresses quickly, and there are generally no obvious symptoms in the early stage. Most patients in China have already progressed to the middle or late stage at the time of diagnosis, and therefore have lost the opportunity to surgically remove the tumor. Palliative treatment is usually adopted for patients with HCC who cannot be operated. Liver tissues of HCC patients contain more water compared to normal liver tissues. Therefore, liver cancer tissues of HCC patients are more sensitive to temperature. Therefore, in addition to surgical treatment, ultrasound-guided microwave ablation is a common method used clinically to treat HCC. After the microwave ablation needle is placed into the tumor lesion, the water in the lesion is evaporated by the warming effect of microwave, which causes coagulative necrosis of the tumor tissue and thus serves to inactivate the tumor.

Based on the above background, this study will evaluate the safety and effectiveness of ultrasound-guided microwave ablation in patients with HCC through retrospective analysis. This study will follow a clinical research method with consecutive enrollment. The overall survival rate, objective tumor remission rate, serum index, and incidence of adverse reactions will be compared. This study aims to explore the clinical efficacy of ultrasound-guided microwave ablation in the treatment of HCC, in order to provide a reference for clinical practice.

## 2 Methods

### 2.1 Study design

**Figure 1:**
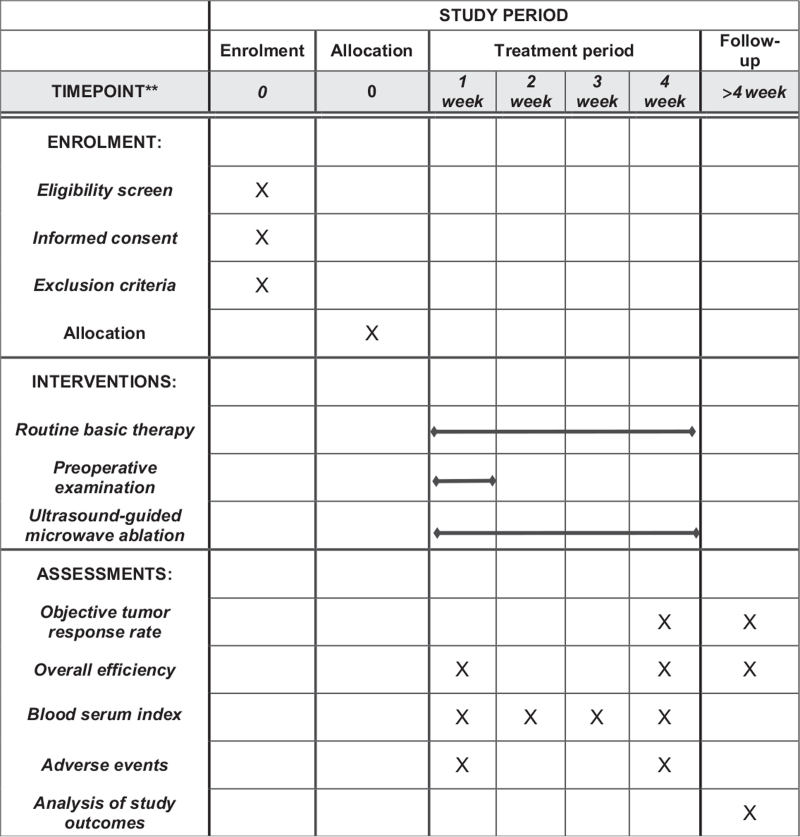
SPIRIT figure for the schedule of enrollment, interventions, and assessments.

This will be a retrospective, single-blind clinical observational tiral to evaluate the efficacy and safety of ultrasound-guided microwave ablation as an adjunctive therapy for patients receiving HCC. All ligible individuals (60) will be randomly assigned to the observation group (n = 30) or the routine group (n = 30) in a 1:1 ratio. This study protocol will be approved by the Ethics Committee of the Second Affiliated Hospital of Shaanxi University of Chinese Medicine and registered in the Chinese Clinical Trial Registry (ChiCTR2100052107). This study will not start recruiting participants until approval by the ethics committee. This study protocol conforms to the Standard protocol Items: Recommendations for Interventional Trials (SPIRIT) guidelines.^[7]^ Before randomization, all eligible participants will be asked to sign an informed consent. The flowchart of the study is shown in Fig. 1.

### 2.2 Study subjects

The cases observed in this trial will come from the Department of Hepatobiliary Surgery. And the inpatients diagnosed as HCC by modern medicine. Researchers will evaluate the included cases according to the following criteria:

1. Primary liver cancer is diagnosed by pathological diagnosis or medical imaging examination;
2. Child-Pugh classification of liver function is A and B;
3. Occurrence The number of liver tumors is less than or equal to 3, and there are no signs of internal and external liver metastasis and vascular invasion;
4. Those who have signed the informed consent.

Participants who have 1 or more of the following criteria will be excluded:

1. Those suffering from severe diseases such as malignant hypertension, severe arrhythmia, acute myocardial infarction, and cerebrovascular accident.
2. Patients with recent acute infections, trauma, surgery, diabetic ketoacidosis, and hyperosmolar coma.
3. Patients with severe primary diseases such as brain, kidney and hematopoietic system, mental illness and cognitive dysfunction.
4. Patients with other malignant tumors and other organ failure.

### 2.3 Randomisation, allocation concealment and blinding

The randomization system used for this study will be provided by the Second Affiliated Hospital of Shaanxi University of Chinese Medicine. Eligible patients will be identified by the treating physician based on inclusion and exclusion criteria. Participants will be referred to a study coordinator who will randomly assign them to each group. The randomization list will be kept by the statistician and study coordinator until the end of the study to ensure allocation concealment.

### 2.4 Intervention

#### 2.4.1 Observation group

Take the treatment of ultrasound-guided microwave ablation alone.

1. Preoperative preparation: perfect blood, urine and stool routine examinations; perfect heart, liver, lung, and kidney function tests; perfect coagulation function tests; perfect tumor marker tests; perfect hepatitis B, hepatitis C, HIV, syphilis, etc. an examination. If the patient has hepatitis B or C, check the virus quantitative; perfect blood glucose measurement and electrocardiogram. The main imaging methods for diagnosing primary liver cancer are color Doppler ultrasound, liver-enhanced CT and enhanced MRI, and it is best to take the imaging examination within 1 month before ultrasound-guided microwave ablation. For patients with high clinical suspicion of primary liver cancer, and laboratory examinations found that AFP >400 ug/L, but the above-mentioned imaging examination failed to find the tumor lesions, DSA examination can be done. Eating, drinking, and water were banned for 6 hours before surgery.
2. Ultrasound-guided microwave ablation operation techniques and steps: Determine the site of puncture according to the ultrasound examination results. Under ultrasound guidance, the appropriate angle of needle insertion is selected and the ablation area should cover more than 5 mm of the lesion edge, and the corresponding single-point, multi-point or multi-point superimposed ablation method is selected according to the diameter and number of lesions. The ablation time was set at about 4 to 15 minutes until the tumor tissue and 0.5 to 1.0 cm of liver tissue at its edge were covered by strong echogenicity. The needle was withdrawn at the end of ablation, and the puncture site was treated and bandaged with pressure.

#### 2.4.2 Routine group

Participants who are assigned to the control group will receive other conventional treatment methods. Including gamma-knife and other treatment methods. The gamma-knife procedure is: let the patient lie on the gamma-knife stereotactic radiotherapy system and fix it according to the body position. 5 mm thick CT scans the lesion area continuously. After obtaining the positioning image, under the guidance of the three-dimensional reconstruction image, use the three-dimensional planning system to define the dense tumor area, clinical target area and planning target area. After the physicist designs and plans the target points, he performs multi-target irradiation according to the actual size of the lesion. The dose and time division of the drug used in treatment should be determined according to the tumor size, location, patient's condition and tissue radiosensitivity.

### 2.5 Outcomes

At 1 month after the completion of treatment, we will compare the tumor ablation rate of the 2 groups by enhanced CT or contrast-enhanced ultrasonography (CEUS). In which the tumor lesion is completely covered by the ablation area without residual and neoplastic lesions, it will be regarded as complete ablation; if there is blood flow signal inside the lesion or abnormal enhancement residual area around it, it will be regarded as incomplete ablation. The complications of both groups at 3 months after treatment will be observed to assess their safety, and postoperative follow-up will be performed for 3 months to count the tumor recurrence rate in both groups.

We will compare the specific treatment effects of the 2 groups of patients. We will evaluate the efficacy of tumor treatment according to the mRECIST standard^[8]^: Complete response (CR) refers to the absence of enhancement in all tumors; partial response (PR) refers to the sum of the maximum diameters of enhancement of all tumors Reduce by at least 30% and maintain this state for at least 4 weeks; Progressive disease (PD) refers to the increase in the sum of the maximum diameter of all tumors by at least 20% or the appearance of new lesions; Stable disease (SD) Refers to cases between PR and PD. The objective response rate is equal to (CR+PR)/total number of cases × 100%, and the disease control rate is equal to (CR+PR+SD)/total number of cases × 100%.

### 2.6 Safety

All participants will have some tests to check their body at screening and after 4 weeks of treatment. These tests will be relative to the heart, liver, kidney and other organs, including white blood cell count, haemoglobin, platelets, blood urea nitrogen, erythrocyte sedimentation rate and electrocardiogram. Adverse events (AEs) are defined as any unexpected unfavourable signs, symptoms or feelings occurring in subjects during the entire period, whether or not associated with ultrasound-guided microwave ablation treatment. Every AE will be fully recorded on the case report form, including all details of AEs. Serious AEs that threaten a participant's life will be reported to the Research Ethics Committee concerned within 24 hours.

### 2.7 Data management

Before being included in the medical record, all researchers will be required to participate in a training class to ensure that they strictly abide by the trial protocol and fully comply with the trial management process. The data will be carefully collected and recorded in the case report form. All data will be input to the computer for storage. And after entering the data, a dedicated researcher will conduct 2 checks. Data quality will be regularly checked by research assistants and supervised by monitoring staff.

### 2.8 Statistical analysis

All analyses will be based on established treatment principles and plans. Each group will provide descriptive statistics as the mean change (standard deviation, 95% confidence interval). We will use SPSS statistical software version 24.0 (IBM SPSS Statistics, NY) to analyze all statistical data. *P* value <.05 (two-sided) will be considered statistically significant. We will use analysis of variance (ANOVA) or χ^2^ test to tabulate and evaluate demographic and baseline data. In order to compare the means between the 2 groups, we will use an independent sample *t* test.

## 3 Discussion

More than 60,000 people die of HCC every year worldwide.^[9]^ Chronic viral hepatitis and alcoholic liver disease are the most common causes of liver cirrhosis in China, Europe and the United States, and up to 90% of patients with liver cirrhosis will eventually develop into primary liver cancer. Statistics from the Chinese Center for Disease Control and Prevention show that 90 million people are infected with hepatitis B virus, and there are about 7.6 million people who are carriers of hepatitis C virus. Despite the continuous advancement of liver cancer diagnosis technology and level, there are still nearly two-thirds of HCC patients who are at an advanced stage at the time of diagnosis, which greatly increases the difficulty of treatment.^[5,10]^ The treatment of HCC needs to fully evaluate the patient's general behavioral status, liver function status, and local tumor status. Achieve the best balance between these 3. In the past few decades, the development of the emerging field of interventional oncology has introduced a variety of minimally invasive, safe and effective treatments for liver cancer patients, thereby further expanding the range of desirable treatment options. Ultrasound-guided microwave ablation is recommended as the standard treatment for mid-term HCC, and is recognized by the European Association for the Study of the Liver^[11]^ and the American Association for the Study of Liver Disease^[12]^ guidelines. Ultrasound-guided microwave ablation is a less invasive and faster recovery technique. Ultrasound-guided microwave ablation can be repeatedly applied to multiple, recurrent and larger lesions, and is an important complementary method for the conservative treatment of solid tumors. Based on ultrasound technology to guide the operation, it can improve the effectiveness, accuracy and safety of solid tumor treatment. Compared with other local ablation techniques, Ultrasound-guided microwave ablation is easy to operate, can better control the heating range, has good hemostatic effect, achieves rapid heating and increases the temperature inside tumor cells. In clinical application, all tumors, tissues and their envelopes should be included in the scope of thermal ablation to achieve complete ablation and obtain a “safe margin” to achieve complete tumor destruction.

At present, there is no good plan for the treatment of intermediate and advanced liver cancer. Therefore, it is necessary to explore a combination of multiple treatment methods, such as combined radiotherapy and chemotherapy before or after interventional therapy, molecular targeted drug therapy, immunotherapy, etc. And find the most suitable population for a certain treatment. Based on the above background, this trial will evaluate the safety and effectiveness of ultrasound-guided microwave ablation alone or in combination with other treatment methods in patients with advanced HCC through the method of retrospective analysis. With the continuous development and progress of medical research, more HCC patients will be benefit from it.

## Author contributions

**Conceptualization:** Jing Qi.

**Data curation:** Jing Qi.

**Funding acquisition:** Yong-Li Chang, Lin-Xia Meng.

**Investigation:** Wei Zhong.

**Project administration:** Bing Ran.

**Resources:** Yong-li Chang, Lin-Xia Meng.

**Software:** Min-Na Chen.

**Validation:** Wang Zhang.

**Visualization:** Wang Zhang.

**Writing – review & editing:** Lin-Xia Meng.
